# A comparison of the characteristics of suicide attempters with and without psychiatric consultation before their suicidal behaviours: a cross-sectional study

**DOI:** 10.1186/1471-244X-14-146

**Published:** 2014-05-21

**Authors:** Kohei Harada, Nobuaki Eto, Yoko Honda, Naoko Kawano, Yuma Ogushi, Mayuko Matsuo, Ryoji Nishimura

**Affiliations:** 1 Department of Psychiatry, Faculty of Medicine, Fukuoka University, 7-45-1 Nanakuma, Jonan-ku, Fukuoka 814-0180, Japan

**Keywords:** Emergency medicine, Psychiatry, Psychiatric consultation, Suicide, Suicide attempts, Suicide prevention

## Abstract

**Background:**

Because psychiatric disorders are risk factors for suicide, psychiatric consultation should be an essential element of suicide prevention among individuals with a high risk of suicide. The aim of the present study was to compare the characteristics of individuals who had or had not received psychiatric consultation before they attempted suicide in Japan.

**Methods:**

Clinical records were used to identify 300 consecutive persons who were admitted to the hospital for attempting suicide between April 2006 and March 2013. We divided the patients into two groups. One group consisted of patients who consulted a psychiatrist before their suicidal behaviours (the consultation group), and the other group consisted of patients who had not consulted a psychiatrist before their suicidal behaviours (the non-consultation group). Group differences were analysed with respect to gender, age, method of suicide attempts, psychiatric diagnosis (ICD-10), and duration of hospitalisation in the emergency unit.

**Results:**

Females tended to be over-represented in the consultation group (73.0%), and males tended to be over-represented in the non-consultation group (59.8%). Poisoning by prescription drugs was used more frequently as a method of suicide in the consultation group than in the non-consultation group. Neuroticism and related disorders were higher in the non-consultation group (33.7%) than in the consultation group (18.9%). Mood disorders (32.6%) were nearly as common as neuroticism in the non-consultation group, and together they accounted for almost two-thirds of all diagnoses. Mood disorders were comparable between the consultation group (30.9%) and the non-consultation group (32.6%). Adult personality disorders (13.3%) and schizophrenia and related disorders (26.0%) were higher in the consultation group than in the non-consultation group.

**Conclusions:**

Measures have to be taken to encourage people with these diverse characteristics to consult psychiatrists, and psychiatrists have to regularly evaluate patients for suicide risk. Furthermore, we need further research on the relationship between psychiatric consultation and poisoning by prescribed drugs.

## Background

The annual number of suicides in Japan jumped dramatically from 24,931 in 1997 to 32,863 in 1998 [1], which increased the suicide rate from 19.3 per 100,000 people in 1997 to 26.0 per 100,000 people in 1998. In response to this serious situation, the “Basic Act on Suicide Countermeasures” was enacted in 2005, and the “Comprehensive Measures to Prevent Suicide” was established in 2006. After an interval of 15 years, the annual number of suicides in Japan decreased to 27,858 in 2012.

Psychiatric disorders have been found to be a risk factor for suicide, and systematic literature reviews indicate that more than 80% of individuals who commit suicide are diagnosed with a psychiatric disorder [2,3]. In Japan, one study found that over 80% of individuals who were admitted to a tertiary emergency facility after highly lethal suicide attempts were diagnosed with Axis I psychiatric disorders [4], according to the Diagnostic and Statistical Manual of Mental Disorders (DSM-IV). Thus, psychiatric consultation with patients who have a high risk of suicide could be important for suicide prevention.

However, there are many individuals who attempt suicide even though they have consulted psychiatrists. A review of previous studies reported that approximately 50% of persons who complete suicide have had a prior psychiatric consultation [5]. Determining the characteristics of those people who attempt suicide even though they have a history of psychiatric consultation could provide insight into these problems and suggestions for suicide prevention.

The concept of emergency medicine is different in Japan than it is in Western countries [6,7]. The emergency medicine system in Japan was established to utilise resources for emergency medical services better, by triaging patients depending on the acuity of their problem. There are three different levels of designated Emergency Care in Japan. 1) “Primary Emergency Care”, which serves patients with low-acuity conditions, who can be safely discharged to their homes, 2) “Secondary Emergency Care”, which serves patients with moderate-acuity conditions, who require admission to a general inpatient unit, and 3) “Tertiary Emergency Care”, which serves patients with high-acuity conditions, who require intensive care or emergency surgery. In major metropolitan areas, each medical facility has a designated level of Emergency Care they can provide, and accept patients from the emergency medical system. Emergency department staffing historically has been based on a “Multi-Specialists Model” with Specialist Physicians representing different services.

Our Fukuoka University Hospital is a “Tertiary Emergency Facility”. About 70 patients who exhibit suicidal behaviour are transferred to the Emergency and Critical Care Centre (ECCC) every year. Until 2005, a psychiatrist had a consultation to evaluate patients with suicidal behaviour only when it was requested. Since 2006, we started the suicide prevention liaison service in which psychiatrists in the suicide prevention team evaluate and care for all patients who have suicidal behaviour.

There have been only a few published reports of psychological autopsies of Japanese samples, and their samples have been small [8-10]. Therefore, we conducted the current study to try to obtain a better description of the characteristics of persons who make severe suicide attempts. We thought this information would help to improve our understanding of suicide attempts and suicide prevention for patients who have characteristics similar to suicide attempters. Previous research indicates that medically serious suicide attempters and suicide completers are two overlapping populations that share common characteristics [11]. Moreover, a history of previous suicide attempts is an important high-risk factor for subsequent suicide [12-14], with about 40% of people who commit suicides having a history of suicide attempts [15]. Continued care for those who attempt suicide would be important for suicide prevention, and the World Health Organization (WHO) has proposed an intervention study of medically treated suicide attempters [16].

Since we started the liaison service for individuals who have attempted suicide (suicide attempters) in 2006, we found that there are two groups of suicide attempters. One group consists of those who had consulted a psychiatrist before engaging in suicidal behaviour, whom the liaison service could re-evaluate and provide interventions. The other group consists of persons who had not consulted a psychiatrist before their suicidal behaviour. The liaison service gave them their first psychiatric evaluation. In this study, we examined sociodemographic and psychiatric data from medically serious suicide attempters, with and without a history of psychiatric consultation before their suicidal behaviour. We thought this comparison would provide a better understanding of the characteristics of both groups and give us important information to guide psychiatric treatment and suicide prevention.

## Methods

### Study design

The study used a cross-sectional design to compare patients who had or had not had psychiatric consultation before attempting suicide.

### Study participants

This study was conducted at the ECCC of Fukuoka University Hospital, from April 1, 2006, to March 31, 2012. There are three ECCCs in Fukuoka City (Japan), which has a population of 1.5 million people. During the study period, 430 patients who had engaged in suicidal behaviours were admitted to ECCC.

The suicide prevention group in the department of psychiatry attended regular conferences in the ECCC and discussed all the patients who had suicidal behaviours. If there was a patient who was suspected of having attempted suicide, the psychiatrist determined whether or not he/she had suicidal behaviours. For example, when a patient who fell from a height was admitted to the ECCC, the psychiatrist assessed the following: (1) if the behaviour was intentional; (2) if he had a clear intent to commit suicide; (3) if he employed lethal methods; (4) if he had considered the prospect of lethality; (5) if he had had previous suicidal ideation; and (6) if his suicidal intent was confirmed objectively by a suicide note. We referred to the results of the inquest at the same time. If it was determined the behaviour was suicidal, the patient was evaluated and treated by a psychiatrist in the suicide prevention group. Thus, the 430 patients represent the total number of patients admitted to the ECCC from 2006 to 2012. If someone received a consultation or a diagnosis at a different hospital, we telephoned the hospital and obtained the patient’s referral document from the hospital. We stored the data of those patients who had suicidal behaviours in an electronic database with a security code.

These patients were divided into two categories: (a) patients who were alive at the point of discharge were classified as “suicide attempters” (n = 300); and (b) patients who were dead at the point of discharge were classified as “suicide completers” (n = 130). The 300 suicide attempters underwent psychiatric evaluation.

The 300 suicide attempters (117 men and 183 women) were interviewed and evaluated by the specialist psychiatrists of the suicide prevention team. Data were collected on the attempters’ gender, age, method(s) of suicide attempt, psychiatric diagnosis, duration of hospitalisation in the emergency unit, and their history of consultation with psychiatrists before admission. Psychiatric diagnoses were made by these psychiatrists according to the ICD-10 criteria. Their intent to die was confirmed at least twice.

The psychiatrists in the suicide prevention group tried to interview all of the patients who had suicidal behaviours and as many family members as possible. However, there were some patients who could not be interviewed. It was not possible to interview some patients, for example because their physical condition was too serious or they were not conscious. So, the psychiatrist interviewed their family, if possible. However, some patients were socially isolated and did not have family members. If they could not interview either the patients or their family, the psychiatrists relied on the patients’ referral documents from other hospitals. Finally, if they could not obtain the patients’ referral documents, the diagnosis was coded as “unknown”.

We divided the suicide attempters into two groups, based on the history of their consultation with psychiatric services before their current admission: a consultation group and a non-consultation group. The consultation group consisted of patients who had consulted a psychiatrist before their suicidal behaviour. The non-consultation group consisted of patients who had not consulted a psychiatrist before their suicidal behaviour. Of the 300 suicide attempters, 12 patients (9 men and 3 women) were excluded because we could not clarify their history of psychiatric consultation before admission. Thus, the total sample size for the study was 288 patients.

### Medically serious suicide attempters

We defined medically serious suicide attempters using the criteria of Beautrais et al. [11]. Medically serious suicide attempters were defined as those patients who required hospitalisation for >24 h and met one of the following treatment criteria: (1) treatment in an intensive care unit; (2) surgery under general anaesthesia (patients with superficial cuts not requiring surgical repair were excluded); and (3) extensive medical treatment (beyond gastric lavage, activated charcoal, or routine neurological observations), including antidotes for drug overdoses, telemetry or repeated tests or assessments. In addition, individuals who attempted suicide by methods with a high risk of fatality, specifically, hanging or jumping, who were hospitalised for more than 24 h, but did not meet the preceding treatment criteria, were also included in the group of persons with medically serious suicide attempts. All the study participants were included in these criteria.

### Statistical analysis

The data are presented as frequency counts, percentages, and means ± SDs. The statistical analyses were conducted using SPSS PASW Statistics 18 for Windows (SPSS Inc.). The chi-square test and the t-test were used for comparisons, as indicated below. A probability level of *P* < 0.05 was considered to be statistically significant.

### Ethics

This study is not an intervention study but an observational study. As it is a retrospective study, we did not obtain consent from the patients, but we did take their privacy into the consideration. Otherwise, we opened the research proposal to the public through the website. This study was approved by the ethics committee of Fukuoka University Hospital.

## Results

### Sociodemographic background

Table 1 shows the age, gender and length of hospital stay of the consultation and non-consultation groups. The mean age of patients in the two groups was comparable, with both groups being in their early 40s, on average. Their mean length of hospitalisation was also similar (between 12 and 13 days). Females were significantly more likely to be represented in the consultation group (females = 73.0%) than the non-consultation group (females = 40.2%) (*P* < 0.001).

**Table 1:** Sociodemographic characteristics of suicide attempters

		**Total (N = 288)**		**Consultation (N = 196)**		**Non-consultation group (N = 92)**		**Analysis**	
		**N**	**(%)**	**N**	**(%)**	**N**	**(%)**	**X**^**2**^	**P-value**
Age	<20	23	(8.0)	8	(4.1)	15	(16.3)	12.7	<0.001
	20-29	57	(19.8)	45	(23.0)	12	(13.0)	3.9	<0.05
	30-39	74	(25.7)	56	(28.6)	18	(19.6)		n.s
	40-49	50	(17.4)	42	(21.4)	8	(8.7)	7.1	<0.01
	50-59	31	(10.8)	17	(8.7)	14	(15.2)		n.s
	60-69	30	(10.4)	19	(9.7)	11	(12.0)		n.s
	70-79	15	(5.2)	6	(3.1)	9	(9.8)		n.s
	80-89	6	(2.1)	2	(1.0)	4	(4.3)		n.s
	90<	2	(0.7)	1	(0.5)	1	(1.1)		n.s
	Mean	41.6 ± 17.8		40.42 ± 21.5		44.14 ± 15.7			n.s
Gender									
Male		108	(37.5)	53	(27.0)	55	(59.8)	28.6	<0.001
Female		180	(62.5)	143	(73.0)	37	(40.2)		
Hospital stay (days)	Mean	12.7 ± 9.5		12.9 ± 14.4		12.2 ± 11.2			n.s

### Methods of suicide attempt

The two groups differed in regard to the methods of suicide attempts (Table 2). The most common methods of attempted suicide in the consultation group were poisoning by prescribed drugs (40.8%), followed by jumping from a height (24.0%), and the use of cutting/piercing instruments (12.8%). The most common methods in the non-consultation group were jumping from a height (19.6%), followed by the use of cutting/piercing instruments (18.5%), and hanging (15.2%).

**Table 2:** Suicide methods of suicide attempters

	**Total (N = 288)**		**Consultation group (N = 196)**		**Non-consultation (N = 92)**		**Analysis**	
	**N**	**(%)**	**N**	**(%)**	**N**	**(%)**	**X**^**2**^	**P-value**
Methods								
Non-violent methods	142	(49.3)	106	(54.1)	36	(39.1)	5.6	<0.05
Violent methods	146	(50.7)	90	(45.9)	56	(60.9)		
Non-violent methods								
Poisoning by prescribed drugs	90	(31.3)	80	(40.8)	10	(10.9)	26.1	<0.001
Poisoning by market drugs	4	(1.4)	3	(1.5)	1	(1.1)		
Poisoning by pesticides	21	(7.3)	14	(7.1)	7	(7.6)		
Poisoning by chemicals	4	(1.4)	2	(1.0)	2	(2.2)		
Poisoning by detergent	10	(3.5)	5	(2.6)	5	(6.5)		
Poisoning by gas	13	(4.5)	2	(1.0)	11	(12.0)		
Violent methods								
Jumping	65	(22.6)	47	(24.0)	18	(19.6)		
Cutting/piercing	42	(14.6)	25	(12.8)	17	(18.5)		
Hanging	27	(9.4)	13	(6.6)	14	(15.2)		
Drowning	6	(2.1)	2	(1.0)	4	(4.3)		
Burning	6	(2.1)	3	(1.5)	3	(3.3)		

We defined poisoning as a non-violent method and the other methods as violent methods, similar to the definition used by Dumais et al. [17]. Patients in the consultation group used non-violent (54.1%) and violent methods (45.9%) about equally, whereas patients in the non-consultation group were more likely to use violent (60.9%) than non-violent (39.1%) methods. These differences between the groups were statistically significant (*P* < 0.05). Poisoning by prescribed drugs was used significantly more frequently in the consultation group (40.8%) than in the non-consultation group (10.9%) (*P* < 0.001).

### Psychiatric diagnosis

Table 3 shows the distribution of psychiatric disorders among the consultation and non-consultation groups. A total of 278 participants (96.5%) met the criteria for an ICD-10 diagnosis, and there were differences between the groups in ICD-10 diagnoses. The patients in the consultation group were most likely to have mood disorders (F3, 30.1%), followed by schizophrenia, schizotypal and delusional disorders (F2, 26.0%), neurotic, stress-related and somatoform disorders (F4, 18.9%) and disorders of adult personality and behaviour (F6, 13.3%). Patients in the non-consultation group were nearly equally likely to have mood disorders (F3, 32.6%) and neurotic-related disorders (F4, 33.7%), which together accounted for about two-thirds of all the diagnoses in this group. Adult personality disorders (F6) and schizophrenia-related disorders (F2) were significantly higher in the consultation group than in the non-consultation group (*P* < 0.01). Neurotic-related disorders (F4) were significantly higher in the non-consultation group than in the consultation group (*P* < 0.01).

**Table 3:** Diagnosis of suicide attempters (ICD-10)

	**Total (N = 288)**		**Consultation group (N = 196)**		**Non-consultation group (N = 92)**		**Analysis**	
	**N**	**(%)**	**N**	**(%)**	**N**	**(%)**	**X**^**2**^	**P-value**
Diagnosis								
Organic, including symptomatic, mental disorders (F0)	9	(3.1)	1	(0.5)	8	(8.7)		n.s
Mental and behavioural disorders due to psychoactive substance use (F1)	16	(5.6)	12	(6.1)	4	(4.3)		n.s
Schizophrenia, schizotypal and delusional disorders (F2)	59	(20.5)	51	(26.0)	8	(8.7)	11.5	<0.01
Mood disorders (F3)	89	(30.9)	59	(30.1)	30	(32.6)		n.s
Neurotic, stress-related and somatofom disorders (F4)	68	(23.6)	37	(18.9)	31	(33.7)	7.6	<0.01
Disorders of adult personality and behaviour (F6)	29	(10.1)	26	(13.3)	3	(3.3)	6.9	<0.01
Disorders of psychological development (F8)	8	(2.8)	5	(2.6)	3	(3.3)		n.s
Others	10	(3.5)	5	(2.6)	5	(5.4)		n.s

### Last consultation before suicide attempts in the consultation group

Table 4 shows the time of the last consultation with psychiatrists prior to suicide attempts in the consultation group. The last psychiatric consultation of 72.4% of patients was within 1 month of admission to the ECCC.

**Table 4:** Last consultations with psychiatrists before suicide attempts in the consultation group

	**Consultation group (N = 196)**	
	**N**	**(%)**
<1 week	71	(36.2)
1 week-1 month	71	(36.2)
1-3 months	20	(10.2)
3-6 months	7	(3.6)
6 months-1 Year	6	(3.1)
> 1 Year	13	(6.6)
Unknown	8	(4.1)

## Discussion

Regarding suicide, there are three types of populations, including (1) suicide completers, (2) medically serious suicide attempters, and (3) self-harm patients. We believe that suicide completers and self-harm patients are different populations, and that medically serious suicide attempters are a population with intermediate characteristics of both suicide completers and self-harm patients. We think suicide completers and medically serious suicide attempters are not the same population, but they are similar and overlapping populations. Fukuoka University Hospital is a “Tertiary Emergency Facility” in Japan. “Tertiary Emergency Care” serves patients with high-acuity conditions, who require intensive care or emergency surgery. For example, patients who poisoned themselves needed intensive care, such as mechanical ventilation with tracheal intubation, or they had severe disturbances of consciousness. Of the 430 patients who were admitted to the ECCC because they had engaged in suicidal behaviours, 300 patients were alive at the point of discharge and 130 patients were dead at the point of discharge. This means that the mortality rate of patients admitted to the ECCC who had engaged in suicidal behaviours was 30%. The severity of the medical conditions of our participants implies that the characteristics of the suicide attempters in our ECCC are similar to those of suicide completers.

### Rate of psychiatric consultation and last psychiatric consultation before attempting suicide

Since a history of previous suicide attempts is the most important risk factor for suicide, the patients in our study all had a high risk of suicide. Our finding that more than 95% (96.5%) of suicide attempters had some psychiatric diagnosis, is consistent with other reports that have found that more than 80% of suicide victims had a diagnosis of at least one mental disorder [2,3]. Our results are also consistent with a study that found more than 80% of suicide attempters who were admitted to tertiary emergency facilities in Japan had mental disorders [4]. The prevalence of psychiatric disorders among the patients in our study puts them at an even greater risk for suicide.

Sixty-eight percent of suicide attempters in our study had consulted a psychiatrist before their suicidal behaviours. This was equivalent to the data of the other two studies about suicide attempters in tertiary emergency facilities in Japan, and the proportion with a previous psychiatric history was about 70% in both studies [4,18]. Although we could not obtain detailed information about the content of consultations in this study, it was surprising that more than 70% (72.4%) of the last consultations were within 1 month of admission to the ECCC. Hence, psychiatric consultation was clearly not enough to prevent suicide attempts, and we need to explore interventions to prevent suicide to replace conventional methods in the future.

### Relationships between suicide prevention strategies and characteristics of the groups

There are three main levels of suicide prevention strategies in the WHO’s “Public Health Action for the Prevention of Suicide—A Framework” [19]: (1) prevention strategies at the general population level; (2) prevention strategies for vulnerable subpopulations at risk; and (3) prevention strategies at the individual level. Prevention strategies at the general population level include not only information campaigns for suicide prevention and mental health, but also: (a) restricting access to the means of self-harm/suicide; (b) developing policies to reduce the harmful use of alcohol related to suicide; and (c) assisting and encouraging the media to follow responsible reporting practices regarding suicide. Prevention strategies for vulnerable subpopulations at risk include: (a) gatekeeper training; (b) mobilising communities; and (c) support for survivors of suicide. Prevention strategies at the individual level include: (a) identification and treatment of mental disorders; and (b) management of persons who have attempted suicide or are at risk.

As the patients in the consultation group attempted suicide even though they had consulted psychiatrists before their suicidal behaviours, the characteristics of the consultation group might have implications for prevention strategies at the individual level. Because the patients in the non-consultation group are those who attempted suicide without psychiatric consultation, there is a possibility that the characteristics of the non-consultation group might have implications for prevention strategies at the general population level, as well as prevention strategies for vulnerable subpopulations at risk, including information campaigns and gatekeeper training.

### Characteristics of the consultation group

Suicide attempters who had consulted a psychiatrist before their suicidal behaviours differed from those who had not in a number of ways. For example, the percentage of females in the consultation group was significantly higher than in the non-consultation group (*P* < 0.001). This may be because females tend to pursue help-seeking behaviour, such as psychiatric consultation, more than males do [20]. Nevertheless, psychiatric consultation apparently was not enough to prevent females from attempting suicide. Clearly, psychiatric consultation is not enough to prevent suicide, so psychiatrists have to evaluate a patient’s risk of suicide and provide intensive interventions designed to prevent female suicides.

Adult personality disorders and schizophrenia-related disorders were higher in the consultation group than in the non-consultation group (*P* < 0.01). Psychiatric consultation might be expected to be less effective in preventing suicide among these kinds of patients than other patients. Hence, it is particularly important to include an evaluation of suicide risks for these patients and to use hospitalisation as a crisis intervention to examine these patients.

Poisoning by prescribed drugs was more frequent in the consultation group than in the non-consultation group (*P* < 0.01), with 40% of the consultation group employing prescribed drugs to attempt suicide. We presume that this is related to the availability and accessibility of prescription drugs. Hence, restricting access to certain classes of prescription drugs can be a valuable suicide prevention measure [21]. Barbiturates, paracetamol, tricyclic antidepressants (TCAs), neuroleptics, and other drugs are regarded as fatal drugs when they are used as poisons [22], so limiting access to these potentially fatal drugs might be especially important for patients with a high suicide risk.

### Characteristics of the non-consultation group

The percentage of males in the non-consultation group was higher than in the consultation group (*P* < 0.001), which suggests the possibility that males do not consult with psychiatrists even though they have psychiatric disorders or suicidal ideation. Information campaigns and other measures that promote psychiatric care for at-risk individuals are needed, especially for males.

Neurotic, stress-related and somatoform disorders were higher in the non-consultation group than in the consultation group (*P* < 0.01). There is evidence that suggests that most depressed patients go to primary care services, but their depression might not be recognised [23]. Screening and evaluating for depression and its suicide risks in primary care settings would be important for suicide prevention.

Patients in the non-consultation group were significantly more likely to use violent methods than patients in the consultation group. Since there were more males than females in this group, and males are more likely to employ violent methods of suicide, we analysed the data by gender. Although males were somewhat more likely (65.5%) to use violent methods than females (54.1%), this sex difference was not statistically significant.

Restricting access to those violent methods of suicide could be important for preventing suicides of those who do not consult with psychiatrists. Moreover, taking actions to help prevent re-attempts of suicide attempters who come to emergency departments is very important because it is the first chance to provide these individuals with intensive interventions, especially those who had not consulted with psychiatrists in the past.

A previous study of patients with severe depression indicated that severe suicide attempts appear to be predictive for suicide in males [24]. These are similar to the characteristics of the non-consultation group in the present study.

### Problems and proposals for suicide prevention

We should point out some problems for suicide prevention that are indicated by our study’s results. The first problem entails the characteristics of the two groups. Patients in the non-consultation group were more likely to be male, and to have psychiatric diagnoses of mood and neurotic disorders. The second is the problem of poisoning by prescribed drugs in the consultation group. The fact that drugs prescribed for treatment were used as a method of suicide presents complicated and ironical problems. The third is that many patients attempted suicide even though they consulted psychiatrists.

We propose four points regarding these problems. First, we need an information campaign and other measures to promote psychiatric consultation to prevent suicides, especially among males. Multilevel and multimodal interventions have been suggested for suicide prevention [25-27]. In addition, we should improve the quality of screening patients and of evaluating their suicide risks in primary care settings [23,28]. We propose that focusing on male patients with Mood and Neurotic disorders should be emphasised as a community-based, suicide prevention strategy. We need further research to examine what strategies are effective for the general population level and for vulnerable subpopulations at risk.

Second, we found that poisoning by prescribed drugs was more frequent in the consultation group than in the non-consultation group. Hence, research should be conducted on the relationship between psychiatric consultation and poisoning by prescribed drugs. We should consider how to manage those drugs for patients having a high suicide risk, such as an appropriate prescription plan, reducing the number of the days prescribed, more frequent psychiatric consultation, and cooperation with their family members to manage the drugs. Moreover, we need measures to restrict drug access to those with a high suicide risk [21,22].

Third, we need to add the evaluation of suicide risks at hospitalisation as a crisis intervention, especially for patients with schizophrenia-related and personality disorders. We should implement a suicide prevention training system for how to assess and deal with patients with a high risk for suicide [29]. Personality disorders are one of the important risk factors of suicide [30]. Previous research shows that, although suicidal behaviour is a more persistent feature among those with personality disorders, their clinical characteristics at the time of a suicide attempt may not differ from those without personality disorders [31]. Schizophrenia and psychotic disorders are also important risk factors for suicide. The main factors to be taken into account when assessing the risk of suicide in patients with schizophrenia are depressive syndromes, suicidal thoughts, and a history of suicide attempts [32-34]. It is recommended that modifiable factors, such as depression should be managed to reduce the suicidality of hospitalised patients with schizophrenia [35].

Fourth, we should reinforce the prevention of re-attempts of suicide when patients are in the ECCC. By intervening in the ECCC, we can re-evaluate patients who had consulted a psychiatrist before their suicidal behaviours, and make a first evaluation of, and provide interventions for, patients who had not consulted a psychiatrist before their suicidal behaviours. Interventions for suicide attempters in emergency care settings were reported in a previous study [21].

### Methodological considerations

The first limitation of our study is that structured interviews were not used to diagnose psychiatric disorders. Although the psychiatrists in the suicide prevention group tried to interview all the patient who had suicidal behaviours, sometimes the psychiatrists could not interview them because their physical condition was too serious or they were not conscious. It also was difficult to perform structured interviews for all patients because of the short hospitalisation in our ECCC. Instead, the psychiatric diagnosis was made by the specialist psychiatrists of our suicide prevention group and we held discussions of the cases about the psychiatric diagnosis. We made an effort to obtain as much information as possible in a way that was appropriate for the context of the ECCC. The second limitation is that the sample size is relatively small, which means our results should be generalised with caution. However, the study sample provides meaningful data for suicide prevention because the sample consists of medically serious suicide attempters with characteristics similar to suicide completers. The third limitation is that we could not examine the suicide attempters who did not participate in our study. Some patients had disturbances of consciousness because of brain damage, so we could not fully interview them, and some were socially isolated and had no family whom we could interview. The fourth limitation is that our study results might be limited to an urban area population, since the study was conducted at a single centre in an urban area. Different results might be expected in a rural area population. Thus, a further multiple-centre study is required to obtain better results for suicide prevention. The fifth limitation is that we did not have information about the history of consultation with general physicians, or with other mental health providers, such as psychologists, social workers, and psychiatric nurses. Though these professionals provide mental health care in Japan, patients usually consult with a psychiatrist at first. The psychiatrist may suggest patients use these other mental health services in psychiatric hospitals or clinics in Japan, depending on the patient’s needs and problems. Thus, consultation with other mental health providers is preceded by consultation with a psychiatrist.

## Conclusions

Since the groups were characterised by differences in gender and the distribution of certain psychiatric diagnoses, we need measures that promote opportunities for people with these characteristics to consult psychiatrists. We also need to establish mechanisms to ensure that psychiatrists regularly evaluate patients for suicide risks. Furthermore, we would like to suggest there is a need to further explore the relationship between psychiatric consultation and poisoning by prescribed drugs.

## Abbreviations

ICD: International Statistical Classification of Diseases and Related Health Problems; WHO: World Health Organization.

## Competing interests

The authors declare that they have no competing interests.

## Authors’ contributions

All authors contributed to study design. KH, NE, YH, NK, OY, MM assessed the patients and participated in data collection. KH and NE analysed the data and wrote the paper. RN supervised and wrote the paper. KH, NE, YH, NK, OY, MM discussed the ideas in the manuscript and contributed to its preparation. All authors read and approved the final manuscript.

## Pre-publication history

The pre-publication history for this paper can be accessed here:

http://www.biomedcentral.com/1471-244X/14/146/prepub
